# Interleukin-6 as Biomarker for Acute GvHD and Survival After Allogeneic Transplant With Post-transplant Cyclophosphamide

**DOI:** 10.3389/fimmu.2019.02319

**Published:** 2019-10-01

**Authors:** Raffaella Greco, Francesca Lorentino, Rosamaria Nitti, Maria Teresa Lupo Stanghellini, Fabio Giglio, Daniela Clerici, Elisabetta Xue, Lorenzo Lazzari, Simona Piemontese, Sara Mastaglio, Andrea Assanelli, Sarah Marktel, Consuelo Corti, Massimo Bernardi, Fabio Ciceri, Jacopo Peccatori

**Affiliations:** ^1^Haematology and Bone Marrow Transplant Unit, IRCCS San Raffaele Scientific Institute, Milan, Italy; ^2^Università Vita-Salute San Raffaele, Milan, Italy

**Keywords:** allogeneic hematopoietic stem cell transplantation, interleukin-6, graft-vs.-host disease, transplant-related mortality, overall survival

## Abstract

**Background:** Although the outcome of allogeneic hematopoietic stem cell transplantation (allo-HSCT) has dramatically improved in the past decade, it is still compromised by transplant-related mortality (TRM), mainly caused by Graft-vs. -Host Disease (GvHD).

**Methods:** We conducted a prospective observational study to ascertain the potential of serum interleukin-6 (IL6) levels, measured before conditioning and 7 days after allo-HSCT, in predicting acute GvHD, TRM and survival after allo-HSCT with Post-Transplant Cyclophosphamide (PT-Cy) based GvHD prophylaxis.

**Results:** Between April 2014 and June 2017, we collected samples from 166 consecutive allo-HSCT patients. By ROC analysis, we identified a threshold of 2.5 pg/ml for pre-transplant IL6 and 16.5 pg/ml for post-transplant IL6. Both univariate and multivariate analyses confirmed the ability of high baseline IL6 levels to predict worse OS (HR 4.3; *p* < 0.01) and grade II–IV acute GvHD (HR 1.8; *p* = 0.04), and of high post-transplant IL6 to identify patients with worse OS (HR 3.3; *p* < 0.01) and higher risk of grade II–IV (HR 5; *p* < 0.01) and grade III–IV acute GvHD (HR 10.2; *p* < 0.01). In multivariate analysis, both baseline (HR 6.7; *p* < 0.01) and post-transplant high IL6 levels (HR 3.5; *p* = 0.02) predicted higher TRM.

**Conclusions:** IL6 may contribute to the risk stratification of patients at major risk for aGvHD and TRM, potentially providing a window for additional prophylactic or preemptive strategies to improve the quality of life in the early post-transplant phase and the outcome of allo-HSCT.

## Background

Allogeneic hematopoietic stem cell transplantation (allo-HSCT) is a curative treatment option for many malignant and non-malignant hematological disorders (1–3), still limited by severe complications and transplant-related mortality (TRM).

Acute Graft-vs.-Host Disease (aGvHD) is a leading cause of morbidity and TRM after allo-HSCT. Despite prophylactic treatment with immunosuppressive agents, historically 20–80% of recipients develop aGvHD after allo-HSCT (4). Post-transplant cyclophosphamide (PT-Cy) has emerged as a promising pharmacological strategy in the setting of allo-HSCT (5–7), thanks to its safety profile and effectiveness in reducing GvHD and finally TRM (6).

New diagnostic and therapeutic tools are still needed to customize the administration of immunosuppressive drugs for patient care optimization. To that end, there has recently been considerable research effort devoted to the discovery and validation of GvHD-relevant biomarkers (8).

The paucity of validated biomarkers for aGvHD is partly because of the complex physiopathology of GvHD that can be considered in a framework of three distinct sequential phases of immune system cellular activation and cytokine production, which would be expected to influence specific cellular and protein levels in patient's blood (8, 9).

Thus, biomarkers that are GvHD and target-organ specific may improve the diagnosis, management, and prognosis of post-transplant complications (8). Potential applications include predicting response to treatment, defining new risk stratification that incorporates biomarker values, and initiating preemptive therapy before onset of clinical symptoms (8).

The balance between pro- and anti-inflammatory cytokines influences the risk of aGvHD. Interleukin-6 (IL6) is a cytokine associated with several inflammatory diseases (10) and a modulator of the immune responses involved in aGvHD pathogenesis (11, 12).

With increasing insight into the complex signaling events induced by IL-6, more specific blockade of the anti-inflammatory functions of IL-6 has been developed to treat autoimmune and neoplastic disorders (12, 13).

In a previous preliminary experience, we analyzed IL6 levels in combination with other biomarkers (ceruloplasmin, cholinesterase, albumin, immunoglobulin A, gammaglutamyltransferase, white blood cells, neutrophils, hemoglobin, platelets, and glycaemia), observing that pre-transplant IL6 levels are able to predict aGvHD and TRM (unpublished data), and paving the way for the current prospective study.

Aim of this study is the early identification of patients at increased risk of HSCT-related complications, with a focus on aGvHD, according to a new potential biomarker, IL6.

We report herein the results of a prospective observational study to ascertain the potential of serum IL6, measured before conditioning and 7 days after allo-HSCT, in predicting main transplant outcomes with PT-Cy.

## Materials and Methods

### Patient and HSCT Procedures

All adult patients were treated according to current Institutional programs upon written informed consent for transplant procedures, the use of medical records for research and for immunological studies.

Patients were affected by high-risk hematological malignancies.

The conditioning regimen was treosulfan-based. All patients received a conditioning regimen based on treosulfan (14 g/m^2^/day) on days −6 to −4 and fludarabine (30 mg/m^2^/day) on days −6 to −2, classified as reduced intensity conditioning (RIC), and nowadays largely considered a full-intensity but reduced-toxicity conditioning regimen (14–16). The majority of patients received an intensified conditioning with the addition of melphalan 70 mg/m^2^/day on days −3 and −2 or thiotepa 5 mg/kg/day on days −3 and −2, classified as myeloablative conditioning (MAC) regimen.

All patients received PT-Cy (50 mg/kg/day) on days 3 and 4 (17, 18). Sirolimus was given from day 5, and withdrawn between months 3 and 6 after HSCT in absence of GvHD or relapse. Mycophenolate mofetil (MMF) was added from days 5 to 30, if the donor was a matched unrelated donor (MUD) or haploidentical donor (mismatched related donor; MMRD). Graft source was predominantly unmanipulated peripheral blood stem cells (PBSCs).

### Study End Points and Definitions

The aim of the present study is to evaluate IL6 as early biomarker to predict the major outcomes and complications (particularly aGvHD) in patients undergoing allo-HSCT.

Clinical and blood IL6 analysis were prospectively conducted on consecutive patients undergoing allo-HSCT with PT-Cy at the Hematology and Bone Marrow Transplant Unit of Ospedale San Raffaele between April 2014 and June 2017.

Acute GvHD was defined and scored assessed following the IBMTR Severity Index and the Glucksberg criteria (19–21).

#### Sample Collection and Analysis

**Figure 1:**
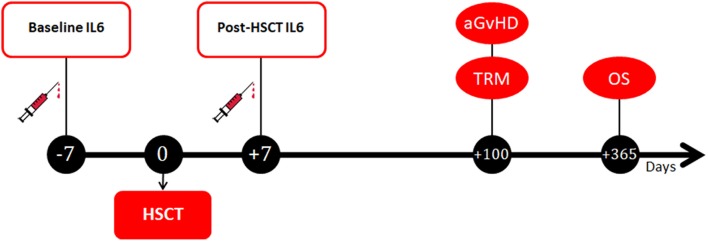
Timepoints of blood samples collection and clinical outcomes assessment. IL6, Interleukin 6; HSCT, hematopoietic stem cell transplantation; aGvHD, acute graft-vs.-host disease; TRM, transplant-related mortality; OS, overall survival.

Peripheral blood samples were collected from patients at two different timepoints (Figure 1). The first sample was collected at baseline, on the day of the initiation of the pre-transplantation conditioning regimen (i.e., 7–14 days before the transplant). The second sample was collected 7 days after the transplant, in correspondence to the period of full aplasia, before engraftment. At each timepoint, after centrifugation samples were stored at −20°C in different tubes until further processing. Serum measurement of IL6 was performed by ELISA assay with the IL-6 Human Instant ELISA™ Kit (BMS213INST, eBioscience) by Thermo Fisher Scientific-Invitrogen and the DSX SER/MET/090 automated ELISA processing system. According to the manufacturer instructions, serum IL6 reference values are set at 0–10 pg/ml.

### Statistics

Categorical variables were described as frequencies and continuous variables as median value.

The Receiver Operating Characteristics (ROC) curve analysis was adopted to identify the optimal cut- off values of baseline and post-transplant IL6 levels for prediction of aGvHD and TRM (22). We transformed these outcomes into binary endpoint (aGvHD at 100 days; TRM at 1 year) and, therefore, only patients who had a minimum of 100 days and 1 year, respectively, of follow-up, or who died within these timeframes were considered in the analysis. Patients experiencing a competing event for aGvHD and TRM were excluded from ROC curve analysis. IL6 levels were then tested on a validation cohort of patients receiving allogeneic HSCT with anti-thymocyte globulin as GvHD prophylaxis.

The Fisher's exact test was performed to determine differences in the frequencies of categorical variables between the two groups defined by the identified cut-off values of baseline and post-transplant IL6 levels. The Mann-Whitney U test was used to determine differences in the median of continuous variables between the two groups (**Tables 2, 3**).

Overall survival (OS) was defined as the interval from allo-HSCT to death whatever the cause, and patients were censored at the date of last contact if alive. TRM was defined as death from any cause while in continuous remission of the primary disease.

Cumulative incidences were estimated for acute GvHD and TRM and to accommodate competing risks (23). Relapse or progression was a competing risk for TRM. Relapse/progression and death from any causes were competing risks for GvHD.

The probability of OS was estimated using the Kaplan-Meyer estimator (24). Log-rank test was used for univariate comparisons of survival curves (25), while the Gray's test was conducted to compare cumulative incidences of competing-risks endpoints (26).

Factors predicting aGvHD and TRM incidence and OS were studied using Cox regression model (27). The variables included in the regression analysis were: patient age (according to median values), Disease Risk Index (DRI) score (28), Sorror-comorbidity index (CI) according to median value (29, 30), type of donor, stem cell source, CMV serostatus and IL6 levels (according to cut-off points derived by ROC analyses). Interactions between each covariate and IL6 levels were tested and not found. The proportional hazard assumption was met for all variables.

A *p*-value of 0.05 was considered significant for determination of factors associated with time to event. Statistical analyses were performed with R (R Development Core Team, Vienna, Austria) software package.

## Results

### Patient and HSCT Characteristics

We collected samples from 166 consecutive adult patients who underwent allo-HSCT with PT-Cy in San Raffaele BMT Unit, between April 2014 and June 2017. Median follow-up on survivors was 469 days (range 69–1,269).

Patient and HSCT characteristics are shown in Table 1. Most patients were affected by myeloid malignancies (AML = 55%, MDS = 14%). According to the Disease Risk Index (DRI) the patients were stratified in low-intermediate (44%), high (44%), and very high (12%).

**Table 1:** Patient and transplant characteristics.

**Patient and transplant characteristics**		
Total number, *n*		166
Patient age y, median (range)		48.5 (15-72)
Patient sex, male (%)		105 (63)
HCT-CI, median (range)		2 (0–7)
Type of diagnosis, *n* (%)	Acute leukemia	104 (63)
	MDS or MPN	31 (19)
	Lymphoma/MM	29 (17)
	Other	2 (1)
DRI at HSCT, *n* (%)	Low-intermediate	74 (44)
	High	74 (44)
	Very high	18 (12)
Conditioning, *n* (%)	MAC	143 (86)
	RIC	23 (14)
Type of donor, *n* (%)	MMRD	89 (53)
	MRD	36 (22)
	MUD	41 (25)
Stem cell source, *n* (%)	PBSC	151 (91)
	BM	15 (9)
Graft content, median (range)	CD34+ cells × 10^6^/kg	5 (1-11)
	CD3+ cells × 10^5^/kg	2046 (164–8061)
H/D CMV status, *n* (%)	Neg/neg	11 (6)
	Neg/pos	8 (5)
	Pos/neg	33 (20)
	Pos/pos	114 (69)

*HCT-CI, Hematopoietic cell transplantation-comorbidity index; MDS, myelodysplastic syndromes; MPN, myeloproliferative neoplasms; MM, multiple myeloma; DRI, Disease Risk Index; MAC, myeloablativeconditioning; RIC, reduced-intensity conditioning; MMRD, mismatched related donor; MRD, matched related donor; MUD, matched unrelated donor; PBSC, peripheral blood stem cells; BM, bone marrow; H/D, host/donor; CMV, cytomegalovirus*.

The majority of patients (91%) received unmanipulated PBSCs. Conditioning was myeloablative in most of the patients (86%). Stem cell donors were MUD (*n* = 41), MMRD (*n* = 89), and matched related donor (MRD; *n* = 36). Post-transplant GvHD prophylaxis was PT-Cy in all patients. Sirolimus and MMF were used as additional prophylaxis (MMF only in MUD and MMRD).

In this population, CI of grade II–IV aGvHD at 100 days was 29% (16% grade III–IV). The median time to aGvHD onset was 30 days (range 11–267), similarly for the RIC and MAC populations. The CI of TRM at 100 days was 8%, with an OS of 70% at last follow-up. Overall, 51 patients died during the follow-up; the primary cause of death was for disease relapse in 27 patients, infections in 15 cases, GvHD in 8 patients and multi-organ failure in one patient. In our cohort of patients, no signs of active infection were present at baseline. At day +7 after transplant, 54% of patients (90/166) showed signs of active infection.

### IL6 and HSCT Outcomes

**Figure 2:**
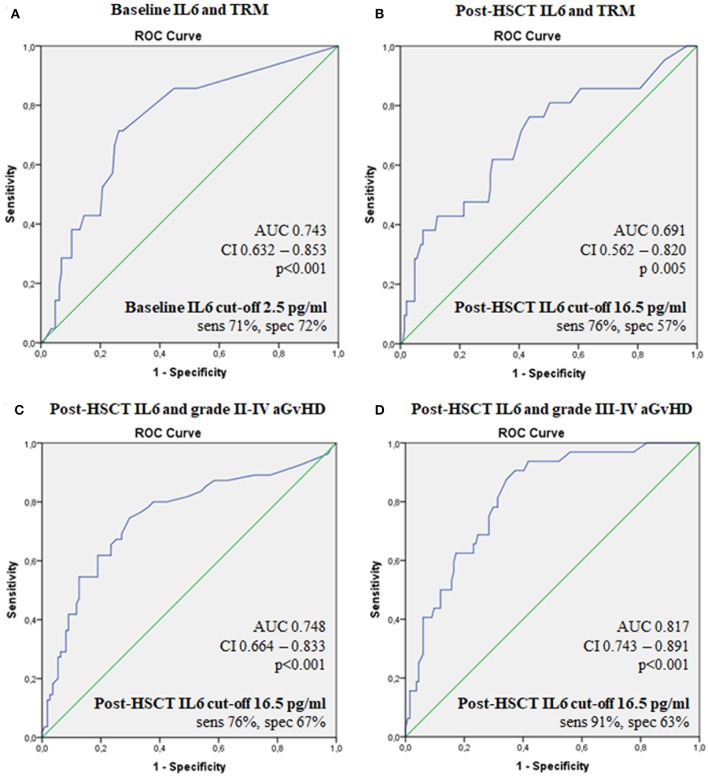
ROC curves for the ability of serum IL6 levels to predict transplant outcomes. Baseline IL6 and TRM **(A)**, post-HSCT IL6 and TRM **(B)**, post-HSCTIL6 and grade II–IV aGvHD **(C)**, post-HSCT IL6 and grade III-IV aGvHD **(D)**. IL6, Interleukin 6; HSCT, hematopoietic stem cell transplantation; aGvHD, acute graft-vs.-host disease; TRM, transplant-related mortality; OS, overall survival; AUC, the area under the ROC curve; CI, 95% confidence interval; sens, sensitivity; spec, specificity.

We identified a threshold (Figure 2) of 2.5 pg/ml for pre-transplant IL6 levels in correlation with TRM (AUC 0.74; sensitivity 71%, specificity 72%, *p* < 0.001) and a threshold of 16.5 pg/ml for post-transplant IL6 as predictor of grade II–IV acute GvHD, grade III–IV acute GvHD and TRM (AUC 0.754, sensitivity 76%, specificity 67%, *p* < 0.001; AUC 0.82, sensitivity 91%, specificity 63%, *p* < 0.01; AUC 0.69, sensitivity 76%, specificity 57%, *p* = 0.005, respectively).

We stratified patients into groups according to whether IL6 concentration was above or below the identified thresholds. Out of 166 patients, 55 patients had baseline IL6 levels higher than 2.5 pg/ml, while 79 patients had IL6 levels higher than 16.5 pg/ml after day +7. Around 67% of patients with high baseline IL6 levels presented IL6 concentrations higher than 16.5 pg/ml at day +7 after transplant, with similar rates between the RIC and MAC populations.

Clinical variables were comparable between the groups stratified according to baseline and post-HSCT IL6 levels (Tables 2, 3), except for DRI score, with a higher percentage of very-high risk patients belonging to group with higher IL6 levels, both at baseline and 7 days after HSCT. Moreover, we found a trend toward high HCT-CI (Hematopoietic Cell Transplantation-Comorbidity Index) in patients with increased IL6 levels, mainly at baseline (Tables 2, 3). We did not see any difference in the distribution of C-reactive Protein (CRP) values according to the identified thresholds of baseline and post-transplant IL6. Moreover, the frequencies of patients with active infections between the two groups of post-IL6 levels, defined according to the threshold of 16.5 pg/mL, was not statistically significant.

**Table 2:** Comparison of patients and transplant characteristics according to pre-HSCT IL6 levels.

	**Pre-HSCT IL6** **<2.5 pg/mL** **(*n* = 111)**	**Pre-HSCT IL6** **≥2.5 pg/mL** **(*n* = 55)**	***p***
Patient age y, median (range)	48 (15-76)	50 (22-77)	0.17
Patient sex, male	66	39	0.17
HCT-CI, median (range)	2 (0–7)	3 (0–7)	0.02
Type of diagnosis, *n*			0.53
Acute leukemia	69	35	
MDS or MPN	22	7	
Lymphoma or MM	19	12	
other	1	1	
DRI at HSCT, *n*			<0.01
Low or intermediate	60	14	
High	45	29	
Very high	6	12	
Conditioning, *n*			0.49
RIC	17	6	
MAC	94	49	
Type of donor, *n*			0.19
MRD	26	10	
MUD	31	10	
MMRD	54	35	
Stem cell source, *n*			0.78
PBSC	100	51	
BM	11	4	
H/D CMV status, *n*			0.94
Neg/neg	8	3	
Neg/pos	5	3	
Pos/neg	23	10	
Pos/pos	75	39	

*HSCT, Hematopoietic stem cell transplantation; HCT-CI, hematopoietic cell transplantation-comorbidity index; MDS, myelodysplastic syndromes; MPN, myeloproliferative neoplasms; MM, multiple myeloma; DRI, Disease Risk Index; MAC, myeloablativeconditioning; RIC, reduced-intensity conditioning; MMRD, mismatched related donor; MRD, matched related donor; MUD, matched unrelated donor; PBSC, peripheral blood stem cells; BM, bone marrow; H/D, host/donor; CMV, cytomegalovirus*.

**Table 3:** Comparison of patients and transplant characteristics according to post-HSCT IL6 levels.

	**Post-HSCT IL6** **<16.5 pg/mL** **(*n* = 87)**	**Post-HSCT IL6** **≥16.5 pg/mL** **(*n* = 79)**	***p***
Patient age y, median (range)	48 (19-71)	48 (15-77)	0.91
Patient sex, male	53	52	0.52
HCT-CI, median (range)	2 (0–7)	3 (0–7)	0.07
Type of diagnosis, *n*			0.56
Acute leukemia	56	48	
MDS or MPN	15	16	
Lymphoma or MM	16	13	
other	0	2	
DRI at HSCT, *n*			0.01
Low or intermediate	47	27	
High	35	39	
Very high	5	13	
Conditioning, *n*			0.11
RIC	16	7	
MAC	71	72	
Type of donor, *n*			0.33
MRD	22	14	
MUD	23	18	
MMRD	42	47	
Stem cell source, *n*			0.79
PBSC	80	71	
BM	7	8	
Graft content, median:			
CD34+ cells × 10^6^/kg	5	5	0.61
CD3+ cells × 10^5^/kg	1,960	1,660	0.82
H/D CMV status, *n*			0.67
Neg/neg	7	4	
Neg/pos	3	5	
Pos/neg	19	14	
Pos/pos	58	56	

*HSCT, Hematopoietic stem cell transplantation; HCT-CI, hematopoietic cell transplantation-comorbidity index; MDS, myelodysplastic syndromes; MPN, myeloproliferative neoplasms; MM, multiple myeloma; DRI, Disease Risk Index; MAC, myeloablativeconditioning; RIC, reduced-intensity conditioning; MMRD, mismatched related donor; MRD, matched related donor; MUD, matched unrelated donor; PBSC, peripheral blood stem cells; BM, bone marrow; H/D, host/donor; CMV, cytomegalovirus*.

Although baseline CRP values correlated with acute GvHD incidence (*p* = 0.001 for grade 2–4 acute GvHD; *p* = 0.002 for grade 3–4 acute GvHD), this association did not affect TRM or OS. On the other hand, CRP levels at +7 days after HSCT were associated only with OS (*p* = 0.04).

**Figure 3:**
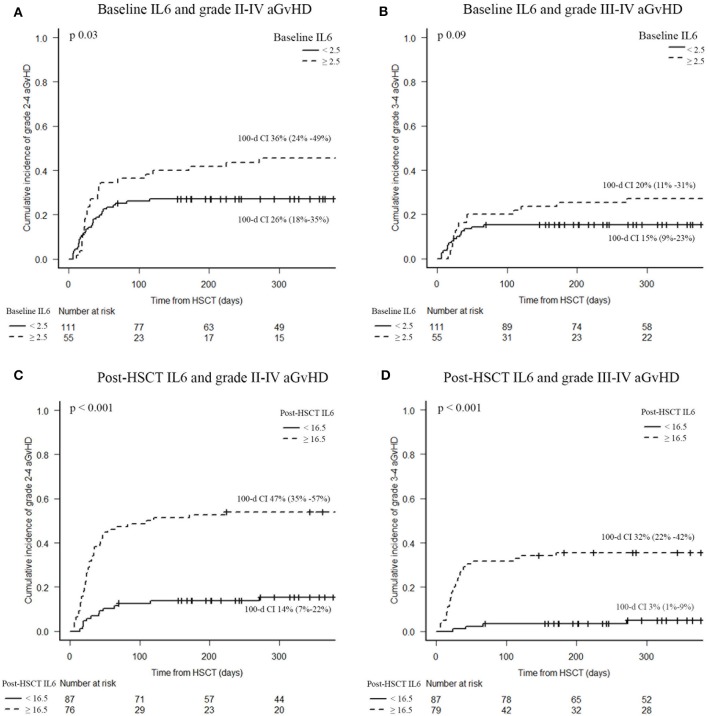
Acute GvHD incidence according to serum IL6 levels. Baseline IL6 and grade II–IV aGvHD **(A)**, baseline IL6 and grade III–IV aGvHD **(B)**, post-HSCT IL6 and grade II–IV aGvHD **(C)**, post-HSCT IL6 and grade III–IV aGvHD **(D)**. CI of acute GvHD were calculated 100 days after HSCT. IL6, Interleukin 6; HSCT, hematopoietic stem cell transplantation; aGvHD, acute graft-vs.-host disease.

Rates of grades II-IV and III-IV acute GvHD were higher in patients with post-transplant IL6 levels higher than 16.5 pg/ml (47 vs. 14%, *p* < 0.001; 32 vs. 3%, *p* < 0.001, respectively), as shown in Figure 3. Instead, baseline IL6 levels higher than 2.5 pg/ml were associated with grade II–IV aGvHD (36 vs. 26%, *p* = 0.03), as shown in Figure 3. In particular, high post-transplant IL6 levels were observed in aGvHD with grade II–IV gut involvement (47 vs. 7%; *p* < 0.001). Moreover, high post-transplant IL6 levels were associated with the development of steroid-refractory aGvHD (28 vs. 2%; *p* < 0.001); around 94% of patients with a steroid-refractory aGvHD showed IL6 levels higher than 16.5 pg/ml at day +7 after transplant.

We found a trend toward a worse TRM in patients presenting high post-transplant IL6 (36 vs. 23%; *p* = 0.06).

**Figure 4:**
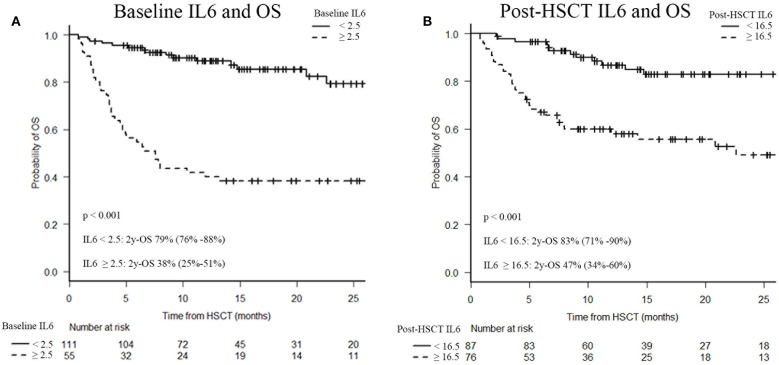
Two-year overall survival (OS) after HSCT according to serum IL6 levels. Baseline IL6 and OS **(A)**, post-HSCT IL6 and OS **(B)**. IL6, Interleukin 6; HSCT, hematopoietic stem cell transplantation; OS, overall survival.

Elevated IL6 concentrations, at baseline and post-transplant, were associated with OS. Indeed, survival analysis confirmed a significantly decreased 2-year OS in patients with baseline IL6 levels higher than 2.5 pg/ml (38 vs. 79%; *p* < 0.001) and/or post-transplant IL6 concentrations higher than 16.5 pg/ml (47 vs. 83%; *p* < 0.001), as shown in Figure 4.

Moreover, relapse incidence was increased in patients with high post-transplant IL6 levels (35 vs. 17%; *p* = 0.03); no correlation was found with baseline IL6 (*p* = 0.23).

Finally, we performed a multivariate analysis, as shown in Tables 4, 5, adjusting for age, Sorror-CI, DRI, donor type, stem cells source and CMV-status. Baseline IL6 concentrations were significantly associated to grade II–IV aGvHD (HR 1.8, 95% CI 1–3.3; *p* < 0.05), TRM (HR 6.7, 95% CI 2.3–20.2; *p* < 0.01), and OS (HR 4.3, 95% CI 2.3–8.1; *p* < 0.01). Instead, post-transplant IL6 levels correlated with grade II–IV aGvHD (HR 5, 95% CI 2.6–9.6; *p* < 0.01), grade III–IV aGvHD (HR 10.2, 95% CI 3.5–29.9; *p* < 0.01), TRM (HR 3.6, 95% CI 1.2–10.5; *p* = 0.02), and OS (HR 3.3, 95% CI 1.7–6.4; *p* < 0.01).

**Table 4:** Multivariate Cox model of pre-HSCT IL6 levels and association with HSCT outcomes.

	**Multivariate Analysis: Baseline IL6**									
	**Grade II–IV aGvHD**		**Grade III–IV aGvHD**		**TRM**		**OS**		**Relapse**	
**Risk factor**	**HR (95% CI)**	***p***	**HR (95% CI)**	***p***	**HR (95% CI)**	***p***	**HR (95% CI)**	***p***	**HR (95% CI)**	***p***
Age ≥ median	0.7 (0.4–1.3)	0.32	0.8 (0.3–1.7)	0.48	1.7 (0.5–5.1)	0.38	1.1 (0.6–2.2)	0.70	0.8 (0.4–1.7)	0.65
HCT-CI ≥ median	1.0 (0.6–1.8)	0.93	1.3 (0.6–2.7)	0.49	1.3 (0.5–3.7)	0.58	1.1 (0.6–2.0)	0.76	0.9 (0.5–1.7)	0.76
DRI										
High vs. low/intermediate	1.1 (0.6–2.0)	0.80	1.1 (0.5–2.5)	0.87	3.1 (0.8–11.2)	0.09	**3.2 (1.4–7.1)**	**0.004**	**2.1 (1–4.6)**	**0.04**
Very high vs. low/intermediate	0.9 (0.4–2.5)	0.93	1.5 (0.5–4.8)	0.49	0.7 (0.1–4.1)	0.66	**2.9 (1.1–7.8)**	**0.03**	**5.2 (1.9–14)**	**0.001**
Donor type										
MRD vs. MMRD	**0.4 (0.2–0.9)**	**0.04**	0.4 (0.1–1.2)	0.10	0.2 (0.02–1.3)	0.09	0.5 (0.2–1.1)	0.09	1.1 (0.5–2.3)	0.84
MUD vs. MMRD	0.5 (0.3–1.1)	0.08	0.6 (0.2–1.5)	0.26	0.5 (0.2–1.9)	0.35	0.5 (0.2–1.2)	0.11	0.9 (0.4–2.1)	0.86
Stem cell source										
PBSC vs. BM	0.98 (0.4–2.3)	0.97	1.6 (0.4–6.9)	0.52	0.5 (0.1–1.8)	0.27	1.3 (0.5–3.8)	0.60	1.5 (0.5–4.2)	0.48
CMV H/D status										
Neg/pos vs. neg/neg	0.6 (0.1–7.1)	0.69	0.9 (0.1–17)	0.97	7.8 (0.4–154)	0.18	4.2 (0.7–25)	0.12	1.3 (0.1–16.2)	0.82
Pos/neg vs. neg/neg	3.1 (0.7–14)	0.15	2.1 (0.2–18)	0.49	1.6 (0.2–17)	0.67	0.8 (0.2–3.1)	0.73	0.9 (0.2–5.1)	0.96
Pos/pos vs. neg/neg	1.7 (0.4–7.1)	0.49	1.6 (0.2–12.5)	0.65	0.66 (0.1–5.9)	0.71	0.8 (0.2–2.9)	0.81	1.8 (0.4–7.6)	0.45
Baseline KPS ≤ 90%	1.5 (0.8–2.9)	0.23	1.9 (0.8–4.7)	0.16	1.3 (0.4–3.9)	0.67	0.7 (0.3–1.3)	0.27	0.5 (0.3–1.1)	0.09
**Baseline IL6 ≥2.5 pg/mL**	**1.9 (1.1–3.6)**	**0.03**	1.7 (0.7–3.8)	0.22	**7.1 (2.3–21.5)**	**0.001**	**4.0 (2–7.7)**	**<0.001**	1.3 (0.6–2.6)	0.50

*HSCT, Hematopoietic stem cell transplantation; aGvHD, acute graft-vs.-host disease; TRM, transplant-related mortality; OS, overall survival; HR, hazard ratio; HCT-CI, hematopoietic cell transplantation-comorbidity index; DRI, Disease Risk Index; MRD, matched related donor; MMRD, mismatched related donor; MUD, matched unrelated donor; PBSC, peripheral blood stem cells; BM, bone marrow; H/D, host/donor; CMV, cytomegalovirus; KPS, Karnofsky Performance Status. Significant values are in bold*.

**Table 5:** Multivariate Cox model of post-HSCT IL6 levels and association with HSCT outcomes.

	**Multivariate Analysis: Post-HSCT IL6**									
	**Grade II–IV aGvHD**		**Grade III–IV aGvHD**		**TRM**		**OS**		**Relapse**	
**Risk factor**	**HR (95% CI)**	***p***	**HR (95% CI)**	***p***	**HR (95% CI)**	***p***	**HR (95% CI)**	***p***	**HR (95% CI)**	***p***
Age ≥ median	0.98 (0.4–2.3)	0.97	1.6 (0.4–6.9)	0.52	0.5 (0.1–1.8)	0.27	1.3 (0.5–3.8)	0.60	1.5 (0.5–4.2)	0.48
HCT-CI ≥ median	1.1 (0.6–1.9)	0.78	1.2 (0.6–2.5)	0.60	1.9 (0.7–4.8)	0.19	1.2 (0.7–2.2)	0.47	0.9 (0.5–1.7)	0.74
DRI										
High vs. low/intermediate	0.9 (0.5–1.8)	0.88	0.8 (0.3–1.9)	0.62	3.3 (0.9–11.7)	0.07	3.8 (1.7–8.6)	0.001	**2.1 (1.1–4.5)**	**0.04**
Very high vs. low/intermediate	0.7 (0.3–1.8)	0.49	1.0 (0.3–3.0)	0.99	1.8 (0.3–10.3)	0.48	5.2 (2–13.6)	0.001	**5.3 (2.1–13.4)**	**<0.001**
Donor type										
MRD vs. MMRD	0.4 (0.2–1)	0.06	0.4 (0.1–1.4)	0.16	0.2 (0.1–1.5)	0.11	0.5 (0.2–1.1)	0.10	1.1 (0.5–2.4)	0.79
MUD vs. MMRD	0.5 (0.3–1.1)	0.08	0.6 (0.2–1.5)	0.26	0.5 (0.2–1.7)	0.29	0.5 (0.2–1.1)	0.08	0.9 (0.4–2.1)	0.81
Stem cell source										
PBSC vs. BM	1.3 (0.6–3.2)	0.49	2.5 (0.6–10.7)	0.22	0.8 (0.2–3.1)	0.78	1.9 (0.7–5.5)	0.22	1.5 (0.5–4.4)	0.43
CMV H/D status										
Neg/pos vs. neg/neg	0.3 (0.1–3.8)	0.36	0.4 (0.1–7.3)	0.52	3.9 (0.2–72)	0.36	3.9 (0.6–25)	0.16	1.4 (0.1–17.1)	0.81
Pos/neg vs. neg/neg	2.7 (0.6–12.2)	0.20	1.8 (0.2–15)	0.59	1.1 (0.1–11.7)	0.91	0.9 (0.2–3.9)	0.93	1.0 (0.2–5.7)	0.95
Pos/pos vs. neg/neg	1.3 (0.3–5.5)	0.72	1.2 (0.1–9.1)	0.88	0.4 (0.1–4.1)	0.46	0.8 (0.2–3.0)	0.78	1.8 (0.4–7.8)	0.44
Baseline KPS ≤ 90%	1.3 (0.7–2.7)	0.39	1.7 (0.7–4.4)	0.25	0.5 (0.1–1.4)	0.17	**0.3 (0.2–0.6)**	**0.002**	0.5 (0.2–0.9)	0.03
**Post-HSCT IL6 ≥16.5 pg/mL**	**5.1 (2.7–9.7)**	**<0.01**	**10.4 (3.5–30.6)**	**<0.01**	**4.4 (1.5–13.5)**	**<0.01**	**4.0 (2–7.7)**	**<0.01**	1.8 (0.9-3.4)	0.07

*HSCT, Hematopoietic stem cell transplantation; aGvHD, acute graft-vs.-host disease; TRM, transplant-related mortality; OS, overall survival; HR, hazard ratio; HCT-CI, hematopoietic cell transplantation-comorbidity index; DRI, Disease Risk Index; MRD, matched related donor; MMRD, mismatched related donor; MUD, matched unrelated donor; PBSC, peripheral blood stem cells; BM, bone marrow; H/D, host/donor; CMV, cytomegalovirus; KPS, Karnofsky Performance Status. Significant values are in bold*.

Further independent prognostic factor for OS was DRI category, while MRD experienced lower hazards for grade II–IV aGvHD, as illustrated in Tables 4, 5. DRI category was the primary prognostic factor for disease relapse.

No interactions were found between DRI score and both baseline and post-HSCT IL6 level thresholds for all endpoints.

### Validation of the Model

To test the predictive accuracy of the new biomarker, we tested it on a retrospective cohort of patients (validation set, *n* = 44), who received allogeneic HSCT with anti-thymocyte globulin as GvHD prophylaxis. To assess uniformity between the training and validation cohorts, we compared patient data between the two populations (Table 6).

**Table 6:** Patient and transplant characteristics of the training and validation cohorts.

		**PTCy-based GvHD prophylaxis**	**ATG-based GvHD prophylaxis**	***p***
Total number, *n*		166	44	
Patient age y, median (range)		48.5 (15-72)	54 (19-70)	0.44
Patient sex, male (%)		105 (63)	30 (68)	0.54
Year of transplant, median (range)		2016 (2014–2017)	2014 (2014–2015)	<0.001
HCT-CI, median (range)		2 (0–7)	2 (0–6)	0.13
Type of diagnosis, *n* (%)	Acute leukemia	104 (63)	24 (54)	0.63
	MDS or MPN	31 (19)	10 (23)	
	Lymphoma/MM	29 (17)	10 (23)	
	Other	2 (1)	0	
DRI at HSCT, *n* (%)	Low-intermediate	74 (44)	25 (57)	0.13
	High	74 (44)	18 (41)	
	Very high	18 (12)	1 (2)	
Conditioning, *n* (%)	MAC	143 (86)	34 (77)	0.15
	RIC	23 (14)	10 (23)	
Type of donor, *n* (%)	MMRD	89 (53)	12 (27)	<0.001
	MRD	36 (22)	0	
	MUD	41 (25)	32 (73)	
Stem cell source, *n* (%)	PBSC	151 (91)	43 (98)	0.13
	BM	15 (9)	1 (2)	
H/D CMV status, *n* (%)	Neg/neg	11 (6)	3 (7)	0.08
	Neg/pos	8 (5)	0	
	Pos/neg	33 (20)	16 (36)	
	Pos/pos	114 (69)	25 (57)	

*HCT-CI, Hematopoietic cell transplantation-comorbidity index; MDS, myelodysplastic syndromes; MPN, myeloproliferative neoplasms; MM, multiple myeloma; DRI, Disease Risk Index; MAC, myeloablativeconditioning; RIC, reduced-intensity conditioning; MMRD, mismatched related donor; MRD, matched related donor; MUD, matched unrelated donor; PBSC, peripheral blood stem cells; BM, bone marrow; H/D, host/donor; CMV, cytomegalovirus; PTCy, post-transplant cyclophosphamide; ATG, anti-thymocyte globulin; GvHD, Graft-vs.-host disease*.

Within this retrospective cohort of patients, the survival analysis confirmed a significantly decreased 2-year OS in patients with baseline IL6 levels higher than 2.5 pg/ml (40 vs. 77%; *p* = 0.001) and/or post-transplant IL6 concentrations higher than 16.5 pg/ml (36 vs. 81%; *p* 0.001).

Rates of grades III–IV acute GvHD were higher in patients with post-transplant IL6 levels higher than 16.5 pg/ml (19 vs. 4%; *p* = 0.05).

High levels of post-transplant IL6 achieved a statistically significant association with worse TRM at 2-year (35 vs. 4%; *p* = 0.009).

## Discussion

There are shortcomings in the prediction of aGvHD, indicating the urgent need for non-invasive and reliable laboratory tests to allow a tailored prophylactic approach.

Timely recognition of patients who are at high risk for aGvHD early in the course of transplantation, may lead to more stringent monitoring, better preventive care, and introduction of alternative and more effective immunosuppressive strategies earlier in the course of treatment (31). In this setting, the use of biomarkers may potentially allow to predict aGvHD before clinical signs appear, predict peak severity of aGvHD before clinical progression, and even identify patients who will not respond to steroids and are at particularly high risk for subsequent morbidity and mortality (31). For the past 20 years, various groups have been investigating potential biomarkers and many have been identified. Nevertheless, no single biomarker or panel of biomarkers has been yet validated for clinical use via large multicenter trials (31–33).

The candidate biomarker of our study was IL6, a cytokine associated with several inflammatory diseases, and a modulator of the immune responses involved in aGvHD pathogenesis (12, 34–36). IL6 can be targeted with a selected inhibitory strategy based on anti-IL6 receptor antibody (10), tocilizumab (TCZ). Moreover, IL6 could be easily and rapidly tested by many centers as routine clinical practice, thanks to the availability of commercial assays. Certainly this represent an important additional value as compared to other proposed biomarkers for GvHD, validated in large clinical trials but still hardly accessible on large scale (37). However, available data on its potential role as systemic biomarker predictive of GvHD are still limited and conflicting (38–44).

We conducted this prospective observational study to ascertain the potential of serum IL6, measured before conditioning and 7 days after allo-HSCT, in predicting aGvHD, TRM and survival after transplant.

We investigated IL6 role in the new transplant setting with PT-Cy. Among 166 consecutive patients who received allo-HSCT with PT-Cy, baseline IL6 levels equal or superior to 2.5 pg/ml identified patients at risk for grade II–IV aGvHD, higher TRM and worse OS. When measured 7 days after HSCT, IL6 levels equal or superior to 16.5 pg/ml were significantly associated with grade II–IV aGvHD, severe aGvHD, higher TRM and lower OS. The correlation between post-transplant IL6 levels and subsequent aGvHD development could be an early index of suboptimal *in-vivo* depletion of allo-reactive T-cell clones. Interestingly, IL6 was also associated with the risk of developing aGvHD with gut involvement and the occurrence of steroid-refractory forms, paving the way for the investigation of IL-6 blockade in prophylaxis and/or treatment of aGvHD with gut involvement and steroid-refractory forms (45–48). Steroid refractory aGvHD is associated with an appreciable morbidity and mortality despite the addition of multiple immunosuppressive agents, and surviving patients often develop chronic GvHD, reducing life expectancy and quality of life (49). Biomarkers, such as IL6, could help to early identify patients who are likely to develop a steroid-refractory aGvHD.

Moreover, IL6 resulted a more reliable predictor of major transplant outcomes in comparison to other biomarkers such as CRP, which conversely appeared a far most non-specific marker, potentially influenced by confounding events. In our analysis, we did not see any difference in the distribution of CRP values according to the identified thresholds of baseline and post-transplant IL6.

In spite of the potential clinical impact of our results, this study has some limitations. The study was limited to patients receiving PT-Cy and sirolimus as GvHD prophylaxis. Although it has the advantage of a homogeneous policy of GvHD prophylaxis, it is based on a single-center experience and limited numbers thus before generalizing our conclusions it is necessary to validate these results in a larger, multicenter study, possibly expanding to patients receiving anti-thymocyte globulin and calcineurin inhibitors. Unfortunately, the design of this study did not include longitudinal samples in the long-term follow-up, preventing us to draw any correlation between IL6 and chronic GvHD.

The timepoints of IL6 measurements were chosen for their clinical relevance in the allo-HSCT course, when there is still the possibility to modify clinical strategies. Baseline IL6 levels may contribute, together with other clinical variables, to modulate the intensity of the transplant strategy, in order to improve final outcomes. Post-transplant IL6, measured when patients are in aplasia and before aGvHD occurrence, should be investigated to early identify patients at risk of severe aGvHD and to provide a window for additional prophylactic and preemptive interventions. Interestingly, a more personalized approach, able to pharmacologically target IL6 by TCZ or Ruxolitinib (46, 50, 51), could be explored also in this setting.

In conclusion, IL6 may contribute to the risk stratification of patients at major risk for aGvHD and TRM, potentially providing a window for additional prophylactic or preemptive strategies to improve the quality of life in the early post-transplant phase and the outcome of allo-HSCT.

## Data Availability Statement

The datasets generated for this study are available on request to the corresponding author.

## Ethics Statement

All patients were treated according to current Institutional programs upon written informed consent for transplant procedures, use of medical records and immunological studies for patients undergoing allogenic HSCT within the non-interventional ALMON study, approved by San Raffaele Institutional Ethical Committee in date 19/10/2007.

## Author Contributions

RG, FL, RN, FC, and JP collected, interpreted the data, and wrote the manuscript. FL performed statistical analysis and prepared figures. RG, FC, and JP designed the study. All authors have approved the final version of the manuscript and contributed to patient clinical care and data collection.

### Conflict of Interest

The authors declare that the research was conducted in the absence of any commercial or financial relationships that could be construed as a potential conflict of interest.
